# Identification of Potential Type II Diabetes in a Large-Scale Chinese Population Using a Systematic Machine Learning Framework

**DOI:** 10.1155/2020/6873891

**Published:** 2020-09-24

**Authors:** Mingyue Xue, Yinxia Su, Chen Li, Shuxia Wang, Hua Yao

**Affiliations:** ^1^Hospital of Traditional Chinese Medicine Affiliated to the Fourth Clinical Medical College of Xinjiang Medical University, Urumqi, China; ^2^College of Public Health, Xinjiang Medical University, Urumqi, China; ^3^The First Affiliated Hospital of Xinjiang Medical University, Urumqi, China; ^4^Center of Health Management, The First Affiliated Hospital, Xinjiang Medical University, Urumqi, China

## Abstract

**Background:**

An estimated 425 million people globally have diabetes, accounting for 12% of the world's health expenditures, and the number continues to grow, placing a huge burden on the healthcare system, especially in those remote, underserved areas.

**Methods:**

A total of 584,168 adult subjects who have participated in the national physical examination were enrolled in this study. The risk factors for type II diabetes mellitus (T2DM) were identified by *p* values and odds ratio, using logistic regression (LR) based on variables of physical measurement and a questionnaire. Combined with the risk factors selected by LR, we used a decision tree, a random forest, AdaBoost with a decision tree (AdaBoost), and an extreme gradient boosting decision tree (XGBoost) to identify individuals with T2DM, compared the performance of the four machine learning classifiers, and used the best-performing classifier to output the degree of variables' importance scores of T2DM.

**Results:**

The results indicated that XGBoost had the best performance (accuracy = 0.906, precision = 0.910, recall = 0.902, *F*‐1 = 0.906, and AUC = 0.968). The degree of variables' importance scores in XGBoost showed that BMI was the most significant feature, followed by age, waist circumference, systolic pressure, ethnicity, smoking amount, fatty liver, hypertension, physical activity, drinking status, dietary ratio (meat to vegetables), drink amount, smoking status, and diet habit (oil loving).

**Conclusions:**

We proposed a classifier based on LR-XGBoost which used fourteen variables of patients which are easily obtained and noninvasive as predictor variables to identify potential incidents of T2DM. The classifier can accurately screen the risk of diabetes in the early phrase, and the degree of variables' importance scores gives a clue to prevent diabetes occurrence.

## 1. Introduction

Diabetes, as a group of metabolic disorders, is characterized by hyperglycemia, which can lead to many serious conditions such as heart disease, kidney disease, vision loss, and lower limb amputation [1]. According to the data from the World Health Organization (WHO), the global epidemic of diabetes currently affects more than 422 million people in 2014 and increased notably in recent decades [2, 3]. In China, the incidence rate of diabetes (100 million of adult patients) was the highest in the world. About 52.7% of diabetes patients have no awareness, and this proposition remains upward [4]. Research has proven that a healthy lifestyle and a reasonable diet structure can effectively delay and prevent the occurrence of type II diabetes mellitus (T2DM) [5]. The American Diabetes Association recommends annual diabetes screening for people over 45 years of age and with major risk factors [6]. China's national plan for the prevention and control of noncommunicable diseases (2012-2015) listed diabetes as one of the key diseases in China and proposed diabetes prediction suggestions based on a blood glucose test and routine physical examination [7].

However, the traditional diabetes screening method needs an expensive blood test and extra manpower, which is a big challenge for the backward remote areas [8]. A diabetes screening model built by easily available indicators, without expensive examinations, is crucial to the occurrence and development of diseases [9, 10].

The analysis of diabetes data is a challenging issue because most of the medical data are nonlinear, nonnormal, correlation structured, and complex in nature [11]. The machine learning (ML) algorithms have dominated in the field of medical healthcare [11–15] and medical imaging for diseases such as stroke, coronary artery disease, and cancer [16–20]. A decision tree (DT) is one of the classical algorithms of ML. This simple and sensitive tree algorithm provides a unique ability to build disease prediction for large datasets [21–23]. Tree embedding algorithms aggregate the results from multiple trees, which usually have better accuracy and generalization ability than a single tree. This includes combining stumps with an enhancement program [24]. The random forest (RF) of a boosting procedure to combine stumps of trees belongs to a “bagging” algorithm [25], which has already been widely used in biological medicine researches [26, 27], especially in the diagnosis of diabetes [11, 12]; AdaBoost with a decision tree (AdaBoost) [28] and an extreme gradient boosting decision tree (XGBoost) [29] belong to “boosting” algorithms, and they had better performance than a decision tree in the prediction and classification [30–32]. In this study, LR- and tree-based models were used. Some studies have confirmed that this method can accurately classify diabetes mellitus [33]. Previous studies have used ML models to classify diabetes. To the best of our knowledge, this is the first diabetes screening model established by comparing four tree-based ML algorithms.

## 2. Methods

### 2.1. Study Population

The national physical examination (NPE) is a free physical examination provided by the Chinese government for all Xinjiang people. The data came from the physical examination data of Urumqi in 2018. A total of 643,439 subjects participated in the examination and signed a written informed consent form. The exclusion criteria of potential participants are the following: (1) pregnancy, (2) people with type I diabetes mellitus (T1DM), and (3) age less than 20 years. Finally, a total of 584,168 subjects from the eligible participants were included in the final analysis. This study was performed in accordance with the principles outlined in the Declaration of Helsinki and approved by the Xinjiang Uygur Autonomous Region CDC ethical committee and the institutional review board.

### 2.2. Diagnosis of T2DM

Subjects with the following criteria were classified as having T2DM: blood glucose 2 hours after meal ≥ 11.1 mmol/l, fasting blood glucose ≥ 7.0 mmol/l, or the main complaint of diabetes and taking hypoglycemic drugs; the final incidence of diabetes was 12.4%.

### 2.3. Baseline Survey

NPE investigates a wide range of lifestyle, dietary, psychosocial, occupational, and biochemical and genetic factors related to the development of chronic diseases. Therefore, the epidemiologists and medical professionals from the CDC in the Uygur Autonomous Region referred to a previous study [34] to design a standard medical examination form, which included 3 parts: a questionnaire, physical examination, and laboratory testing. The examination of all the participants was done by the medical and health teams in the administrative regions, which were made up of full-time employees with medical qualifications and fieldwork experience. In order to ensure the accuracy of the results, all participants were asked to bring their unique national identity (ID) cards and take them as the unique identification. After the investigation, all the data were summarized into the Health Management Hospital of Xinjiang Medical University.

Trained interviewers administered questionnaires during face-to-face interviews. The questionnaires included demographic information, occupational history, socioeconomic status, family and personal disease histories, smoking history, alcohol use, diet, physical activity, and contact history of occupational disease-inductive factors. The physical examinations were performed by trained physicians, nurses, and technicians, in which items included standing height, body weight, waist circumference, heart rate, blood pressure, and abdominal ultrasound. Abdominal ultrasound can observe the shape and size of the abdominal organs; also, it can determine whether these organs have tumors, cysts, or stones, including the liver, kidney, gallbladder, and other organs. For each participant, a 10 ml nonfasting blood sample was collected into three vacuum tubes. The samples were then kept in a portable, insulated cool box with ice packs for up to a few hours before being taken to the local study laboratory for immediate processing. Blood test indicators include blood glucose and blood biochemistry. In this study, we wanted to establish a simple model that can predict the risk of T2DM without blood sampling. We selected 18 variables from the questionnaire and physical examination based on the previous studies [35–37] (Table 1).

### 2.4. Variable Definitions

The potential risk factors in this study to assess T2DM included the following: age, gender, ethnicity, body mass index (BMI), physical activity, smoking, drinking, eating habits, waist circumference, blood pressure, and some comorbidities.

Sociodemographic information included age (years), gender including “male” and “female,” and ethnic groups which were divided into six categories: “Han,” “Uygur,” “Kazak,” “Hui,” “Mongolian,” and “other nationalities”; the baseline comorbidities considered in this study were fatty liver and hypertension (yes or no).

Lifestyle information included smoking, drinking, physical activity, and eating habits. Physical activity was defined as regularly doing at least 20 min per day of physical activity during leisure time over the previous 6 months (yes or no) [38]. Individuals who had been smoking at least one cigarette per day for at least 6 months were defined as smokers, and those who had been drinking alcohol at least once per week for at least 6 months were considered drinkers [39]. We also included daily smoking amount (cigarettes) and weekly drinking amount (“≥170 g” or “<170 g”). Diet habits included 6 options: “meat based,” “meat balanced,” “vegetarian based,” “oil loving,” “sugar loving,” and “salt loving”; participants can choose one or more of them.

### 2.5. Statistical Analysis

The baseline characteristics of the study population were presented as mean ± SD (standard deviation) for continuous normal distribution variables, median (IQR) for continuous nonnormally distributed variables, and number (percentage) for the categorical variables. Differences in variables between diabetes and nondiabetes patients are analyzed by the independent *t*-test for continuous normal distribution variables, the Mann-Whitney test for nonnormally distributed variables, and the chi-square test for categorical variables. All of the tests were two-tailed and considered significant factors whose *p* values were less than 0.05.

### 2.6. Machine Learning System

**Figure 1:**
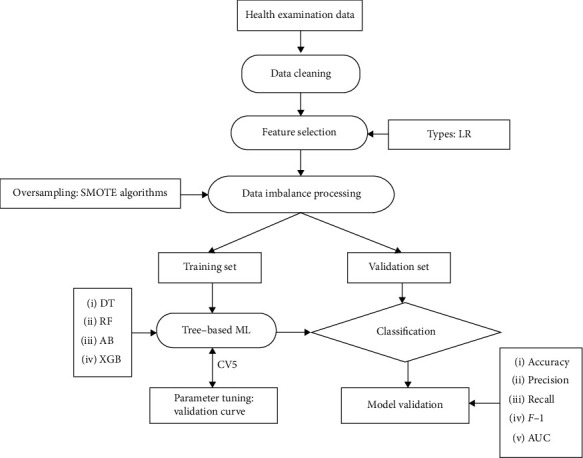
Machine learning flowchart of this study. Abbreviations: LR: logistic regression; DT: decision tree; RF: random forest; AB: AdaBoost; XGB: XGBoost; ML: machine learning.

The major objective of the tree-based ML algorithms is to classify the T2DM. The overview of the proposed tree-based ML algorithms has been shown in Figure 1.

#### 2.6.1. Data Cleaning

NPE data are large and with jumbled variables, with many missing and abnormal values. So data preprocessing is a very important step, and the quality of preprocessing will directly affect the performance of the later prediction model [40]. Firstly, we deleted nearly 200 variables that were not meaningful to this study. Secondly, we filled in outliers and nulls, classification variables were filled with the most frequent value, and continuous variables were filled with a mean value.

#### 2.6.2. Feature Selection

There were some commonly used feature selection techniques in ML/statistics, namely: RF [12, 41], LR [42, 43], mutual information [12, 44], principal component analysis [12, 44, 45], analysis of variance [12, 46], and Fisher's discriminant ratio [12, 44, 47]. In this study, we have used the LR model to identify the risk factor for diabetic disease based on a *p* value (*p* < 0.05) and OR.

#### 2.6.3. Data Imbalance Processing

The number of nondiabetes subjects was greater than the number of subjects with diabetes (an unbalanced-class problem). Generally, classes with few subjects are more difficult to predict than those with numerous subjects [48–51]. We used the SMOTE algorithm to solve the negative impact of class imbalance, which belonged to the method of oversampling; the principle of the method is to increase the number of a few classes of samples in classification to achieve sample balance, and it is widely used because of its ability to preserve important information in samples.

#### 2.6.4. Classifier Comparison

In this study, we used four tree-based ML algorithms: DT, RF, AdaBoost, and XGBoost, all of which were supervised ML methods. DT is a tree structure-based model which describes the classification process based on input features [52]; the advantage of DT is that it is simple and easy to implement, but it often exhibits high variance and overfitting problems, which limits its utility as an independent prediction model. However, it is possible to improve the overall prediction by aggregating the results from multiple trees, which is called the embedding method. RF is one of the common tree embedding methods [53], which uses the bagging method to combine multiple trees. Another ensemble approaches, AdaBoost and XGBoost algorithms [24], use the boosting procedure to combine stumps of trees. These ensemble methods can be loosely conceptualized as forming a robust overall prediction by aggregating the predictions of many simpler predictive models. This is similar to the process of drawing on the advice of many experts to arrive at a clinical diagnosis for a patient, each of whom views the patient in a slightly different way.

#### 2.6.5. Model Evaluation

**Figure 2:**
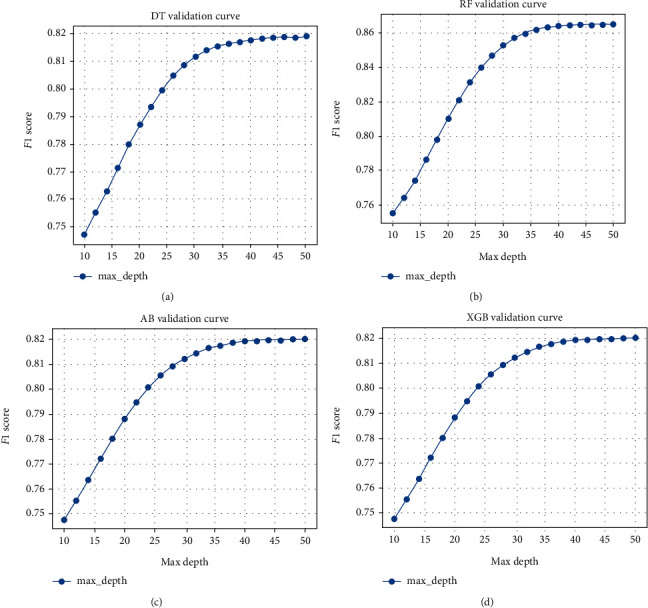
Parameter selection process of the prediction model constructed by four classification tree models: (a) decision tree, (b) random forest, (c) AdaBoost, and (d) XGBoost. Note: the score of *F*‐1 has been tested when the max depth parameter of the model is between 10 and 50.

Balanced datasets were randomly divided into two parts: the training set accounted for 70% of the data and the test set accounted for 30% of the data [21, 54]. In order to improve the accuracy of the classification tree, we have drawn a “verification curve” based on 5-fold cross-validation of four classification trees, and the optimal hyperparameter has been obtained (Figure 2). The algorithms were compared based on a confusion matrix and some indicators including accuracy, precision, recall, *F*‐1, and receiver operating characteristic (ROC) curve. Several important measures, such as accuracy, precision, recall, and *F*‐1, could be calculated by using the confusion matrix.

(1) $$\begin{array}{c} \text{Accuracy}=\frac{\text{TP}+\text{TN}}{\text{TP}+\text{TN}+\text{FP}+\text{FN}},\\ \text{Precision}=\frac{\text{TP}}{\text{TP}+\text{FN}},\\ \text{Recall}=\frac{\text{TP}}{\text{TP}+\text{FP}},\\ F‐1=\frac{2\times \text{Precision}\times \text{Recall}}{\text{Precision}+\text{Recall}}. \end{array}$$

### 2.7. Feature Importance Ranking

Tree-based models can provide measures of variable importance. Unlike the OR values of regression models, ML algorithms cannot estimate an easy explanation number because the relationships that ML algorithms fit are more complex than those of regression models. Therefore, it is not usual to generalize this relationship directly into any one parameter, nor is there a causal relationship or even a statistical explanation [55]. Instead, the measure can often be thought of as rank ordering of which variables are most “important” to the fitted model [56]. Although the variable importance ranking cannot replace the target hypothesis test for a given parameter, it can be used as a means of generating hypotheses to help identify factors that warrant further study, allowing some insight into the factors that most influence the predictions [57].

The software used in this study was Python software version 3.7.2. The “Pandas” library, “NumPy” library, and “Matplotlib” library were used for null and outlier determination and interpolation, the “Imlearn” library was used to solve data imbalance, and the “Sklearn” library was used to establish machine learning models and verify the validation.

## 3. Results

### 3.1. Patients and Variables

A total of 72,027 patients (12.4%) from the pool of 582,438 subjects had T2DM. Each subject was composed of 18 variables (Table 1), including age, BMI, gender, waist circumference, ethnicity, drinking, physical activity, smoking, eating habits, blood pressure, fatty liver, and hypertension. It is observed that all attributes are highly statistically (*p* < 0.001) associated with diabetes.

### 3.2. Feature Extraction Using Logistic Regression

Table 2 shows the effect of the selected factors on T2DM by logistic regression. It was shown that age, BMI, waist circumference, systolic pressure, ethnicity, physical activity, drinking status, weekly drinking amount (g), daily smoking amount (cigarettes), smoking status, dietary ratio (meat to vegetable), diet habit (oil loving), fatty liver, and hypertension are statistically significant factors for T2DM at a 5% level of significance and the rest of the factors are insignificant. These 14 variables were used for tree-based ML algorithms to classify T2DM. Among these statistically significant variables, variables with OR > 1 were the risk factors for T2DM, including age, BMI, waist circumference, systolic pressure, ethnicity (Hui), weekly drinking amount ≥ 170 g, daily smoking amount (cigarettes), smoking status, diet habit (oil loving), fatty liver, and hypertension; variables with OR < 1 were the protective factors, including ethnicity (Kazak and Mongolian), physical activity, drinking status, and diet habit (meat balanced).

### 3.3. Tuning of Parameters

Finally, we got 1,020,822 samples by the SMOTE algorithm (Table 3): 714,575 subjects as the training set and 306,247 subjects as the validation set. The average *F*‐1 score for different models and their parameter are listed in the validation set (Figure 2). When the “maximum depth” of DT takes 44 and that of RF, XGBoost, and AdaBoost takes 40, we got a relatively economical and accurate classification tree model.

### 3.4. Validation of the Training Set

**Figure 3:**
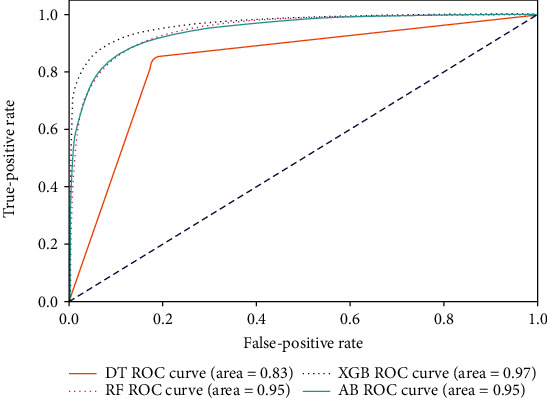
ROC curve of all algorithms. Abbreviations: DT: decision tree; RF: random forest; AB: AdaBoost; XGB: XGBoost.

Our study has built four tree-based ML algorithms.  Table 4 shows the performance of all classifiers. The confusion matrix has been displayed by heatmap; the larger the number, the darker the color of the region, that is, the closer the color of TN and TP regions to orange. On the contrary, the lighter the color of FN and FP regions, the higher the accuracy of the classification model. We got that the result of XGBoost was better than that of the others (accuracy = 0.906, precision = 0.910, recall = 0.902, *F*‐1 = 0.906, and AUC = 0.968).  Figure 3 presents the ROC of all classifiers.

### 3.5. Variable Importance Ranking by XGBoost

**Figure 4:**
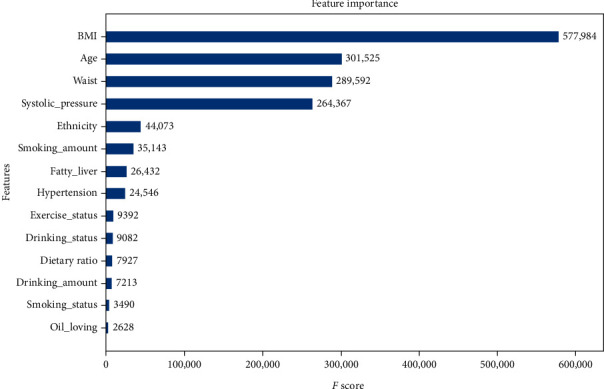
Feature importance contributed to the XGBoost model measured by the *F* score.

In this study, XGBoost was used to rank the LR-selected variables because of its best classification performance. XGBoost provided the importance score of each variable, attributing the predictive risk in 3 ways. Specifically, we chose the default method, which represented the relative number of times a variable is used to distribute the data across all trees. There were only very small differences among the importance scores through the three methods, which did not influence the rank of the variable's impact. The important measurement scores of 14 variables have been shown in Figure 4. BMI is the most significant feature, followed by age, waist circumference, systolic pressure, ethnicity, smoking amount, fatty liver, hypertension, physical activity, drinking status, dietary ratio (meat to vegetable), drink amount, smoking status, and diet habit (oil loving).

## 4. Discussion

In this paper, cases were recruited and consisted of easily acquired variables to establish a screening model for T2DM. LR models were used for selecting the risk factors. Then, we compared the performance of four tree-based ML algorithms (DT, RF, AdaBoost, and XGBoost), and XGBoost got the best performance, which had accuracy = 0.906, precision = 0.910, recall = 0.902, *F*‐1 = 0.906, and AUC = 0.968. Finally, through the best classifier to establish the most important ranking of factors affecting the incidence of diabetes, the results indicate that this strategy successfully achieves accurate and rapid diabetes screening.

The order of feature importance (Figure 3) showed that age, BMI, and waist circumference were the top three influencing factors of diabetes, which was consistent with Pei et al.'s T2MD screening model based on a j48 decision tree [35]. The variables whose OR > 1 are risk factors for the disease, including age, BMI, waist circumference, systolic pressure, hypertension, ethnicity (Hui), daily smoking amount (cigarettes), fatty liver, weekly drinking amount ≥ 170 g, smoking status, and diet habit (oil loving). Xu et al. [36] used the data of the national cross-sectional survey in 2010 for study and found that the risk factors for diabetes were age, smoking, overweight, obesity, dyslipidemia, elevated triacylglycerol, and high systolic blood pressure. Other countries had developed diabetes screening tools, and the American Diabetes Association (ADA) provides a simple “T2DM risk test” that used age, gender, family history of diabetes, hypertension, physical activity, and weight status to assess diabetes risk in the general population [37]. The above conclusions were consistent with the conclusions of this study. Variables with OR < 1 are protective factors, including ethnicity (Kazak and Mongolian), physical activity, weekly alcohol consumption < 170 g, and diet habit (diet balanced). The protective factors include three adjustable indicators, which suggested that people could control the occurrence of the disease through a good lifestyle. Several large-scale trials have demonstrated the benefits of targeted lifestyle interventions to prevent diabetes [58–60].

There are several strengths of our study. First, all the variables come from noninvasive and easily available measurement indexes and questionnaire indexes. The model can be applied to the prediabetes and noninvasive prediction of diabetes without the need for expensive laboratory testing, which is useful, particularly in areas of high epidemiological risk and low socioeconomic status [2, 61].

Second, this study was based on a large Chinese population, with a wide range of population choices and high extrapolation and representativeness. Moreover, our dataset included many major ethnic groups in China, which better evaluated the characteristics of the Chinese population.

Third, in most previous diabetes screening models, smoking and drinking were only divided into two categories (have and have not), so they failed to reflect the impact of frequency and quantity on the disease. Through Figure 3, we knew that compared with the smoking status, the daily smoking amount was more important to the disease. Furthermore, our studies have shown that alcohol was a protective factor for T2DM, but alcohol consumption > 170 g a week increased the risk of diabetes. Previous studies have also confirmed that light-to-moderate alcohol consumption could reduce the risk of T2DM [62, 63]; however, there was a strong dose-response relationship between smoking number, alcohol consumption, and diabetes and cardiovascular disease [64–66], suggesting that while quitting smoking completely and controlling alcohol consumption were our goals, even smoking fewer cigarettes and drinking less alcohol can reduce the risk of the disease.

Fourth, we compared the performance of four tree-based classification models, and XGBoost achieved the best performance. XGBoost used in this study has received extensive attention in recent years due to its excellent learning effect and efficient training speed. XGBoost has more advantages than LR in predicting the occurrence of results rather than measuring the relationship between specific risk factors and events, but its disadvantage is the poor interpretation of risk factors [55]. LR provides a clear interpretation of its coefficients as the odds ratios of the risk factors. We know that the former could get higher prediction accuracy and the latter could get better explanation among variables. In this study, we have first used LR to screen variables and then used XGBoost to classify diseases, which not only improves the accuracy of classification but also gets the risk factors and protective factors for diseases, enlightening us which characteristics may lead to T2DM and which characteristics can prevent T2DM.

Surprisingly, previous studies have found that the course of diabetes is closely related to diet. For example, the Diabetes Prevention Program (DPP) reported that a reasonable diet and exercise can reduce the incidence of type 2 diabetes by 58% [67]. But in this study, we only got the weak effects of meat and vegetable matching and oil preference on T2DM (Figure 3) and did not find that halophilia or sugar addiction is associated with diabetes. However, the effects of these factors on diabetes have been confirmed in previous studies [68, 69]. Eating habits are the main influencing factors of waist circumference and BMI, so we think that diabetes and eating habits are closely related; the possible reasons for the irrelevance might be that the diet survey of Xinjiang national health examination was a cross-sectional study and there was no professional person to evaluate the diet of the physical examination population. The main reason for the error was that the self-reported eating habits of the physical examination population were subjective and professional evaluation indicators are lacking; for this, in the future research, more accurate results can be obtained through the follow-up of people's living habits.

There are several limitations in this study: firstly, since this was a cross-sectional study, we could not assess the causal relationship between T2DM and other comorbidities. Secondly, the data used in this study was the physical examination data of China, which might limit the extrapolation of the results. It is generally believed that there are some differences in the pathophysiology of diabetes between Asians and Caucasians and there are similar differences between Asian countries. Thirdly, previous studies have confirmed that education and family history are also important determinants of diabetes. However, our physical examination data failed to obtain the education and family history of participants. Fourthly, this study only optimizes the “maximum depth” parameter of the classification trees. The machine learning model can improve the performance of the model by tuning multiple parameters, which needs to be further implemented in the future. Finally, some indicators do not have objective and unified evaluation criteria, such as eating habits, which may reduce the accuracy of the prediction model.

## 5. Conclusion

We have proposed a classifier combining tree-based ML algorithms and LR to build a diabetes screening model using 582,438 subjects in China. The ranking of disease risk factors and protective factors provided us with inspiration to prevent diabetes. We also got the dose relationship between smoking and drinking and the disease. In a word, our model can help China's health system to improve the level of early diagnosis of diabetes, suggesting the significance of lifestyle change in the prevention and delay of the disease.

## Figures and Tables

**Table 1:** Characteristics of variables.

Variables	Diabetes (*N* = 72,027)	Nondiabetes (*N* = 510,411)	*p* value
Age (years)	66.43 ± 13.43	52.41 ± 16.06	<0.001
BMI (kg/m^2^)	25.92 ± 3.65	24.37 ± 3.42	<0.001
Waist circumference (cm)	90.20 ± 10.75	84.95 ± 10.71	<0.001
Systolic pressure (mmHg)	130.20 ± 16.52	121.30 ± 14.27	<0.001
Diastolic pressure (mmHg)	77.80 ± 10.56	75.14 ± 9.65	<0.001
Ethnicity, *n* (%)			<0.001
Han	50,691 (70.38)	331,413 (64.93)	
Uygur	10,864 (15.08)	95,913 (18.79)	
Kazak	1147 (1.59)	18,893 (3.70)	
Hui	8126 (11.28)	52,838 (10.35)	
Mongolian	76 (0.11)	1214 (0.24)	
Other nationalities	1123 (1.56)	10,140 (1.99)	
Gender, *n* (%)			<0.001
Male	34,641 (48.09)	239,875 (47.00)	
Female	37,386 (51.91)	270,536 (53.00)	
Physical activity, *n* (%)			<0.001
Yes	26,239 (36.43)	154,585 (30.29)	
No	45,788 (63.57)	355,826 (69.71)	
Drinking status, *n* (%)			<0.001
Yes	15,944 (22.14)	102,852 (20.15)	
No	56,083 (77.86)	407,559 (79.85)	
Drinking amount (g)			<0.001
≥170	6687 (9.30)	39,479 (7.73)	
<170	65,240 (90.70)	470,932 (92.27)	
Smoking amount (cigarettes)	10 (8-20)^∗^	10 (7-20)^∗^	<0.001
Smoking status, *n* (%)			<0.001
Yes	10,683 (14.83)	63,920 (12.52)	
No	61,344 (85.17)	446,491 (87.48)	
Dietary ratio, *n* (%)			<0.001
Meat based	2849 (3.96)	13,554 (2.66)	
Meat balanced	66,603 (92.47)	482,864 (94.60)	
Vegetarian based	2575 (3.58)	13,993 (2.74)	
Sugar loving, *n* (%)			<0.001
Yes	940 (1.31)	4560 (0.89)	
No	71,087 (98.69)	505,851 (99.11)	
Oil loving, *n* (%)			<0.001
Yes	2722 (3.78)	13,068 (2.56)	
No	69,305 (96.22)	497,343 (97.44)	
Salt loving, *n* (%)			<0.001
Yes	4261 (5.92)	20,896 (4.09)	
No	67,766 (94.08)	489,515 (95.91)	
Fatty liver, *n* (%)			<0.001
Yes	22,331 (31.00)	52,800 (10.34)	
No	49,696 (69.00)	457,611 (89.66)	
Hypertension, *n* (%)			<0.001
Yes	29,937 (41.56)	112,348 (22.01)	
No	42,090 (58.44)	398,063 (77.99)	

^∗^Median (IQR). Abbreviation: BMI: body mass index.

**Table 2:** Screening the risk factors for T2DM by multiple logistic regression (CI = confidence interval).

Intercept and variable	Odds ratio	95% CI	*Z* value	*p* value
Age (years)	1.047	(1.046-1.048)	113.625	<0.001
BMI (kg/m^2^)	1.016	(1.012-1.020)	7.894	<0.001
Waist circumference (cm)	1.016	(1.015-1.018)	23.905	<0.001
Systolic pressure (mmHg)	1.002	(1.001-1.003)	5.304	<0.001
Diastolic pressure (mmHg)	1.001	(0.999-1.002)	1.650	0.099
Ethnicity, *n* (%)				
Han	1	Ref	—	—
Uygur	1.011	(0.981-1.043)	0.734	0.463
Kazak	0.460	(0.426-0.497)	-19.669	<0.001
Hui	1.075	(1.040-1.111)	4.269	<0.001
Mongolian	0.464	(0.342-0.616)	-5.127	<0.001
Other nationalities	0.989	(0.912-1.072)	-0.263	0.793
Gender, *n* (%)				
Male	1	Ref	—	—
Female	1.017	(0.994-1.041)	1.444	0.149
Physical activity, *n* (%)				
No	1		—	—
Yes	0.715	(0.699-0.731)	-29.179	<0.001
Drinking status, *n* (%)				
No	1	Ref	—	—
Yes	0.891	(0.864-0.918)	-7.424	<0.001
Drinking amount (g)				
<170	1	Ref	—	—
≥170	1.239	(1.185-1.296)	9.432	<0.001
Smoking amount (cigarettes)	1.005	(1.002-1.007)	3.921	<0.001
Smoking status, *n* (%)				
No	1	Ref	—	—
Yes	1.137	(1.086-1.191)	5.452	<0.001
Dietary ratio, *n* (%)				
Meat based	1	Ref	—	—
Meat balanced	0.917	(0.869-0.969)	-3.105	0.002
Vegetarian based	1.019	(0.941-1.103)	0.455	0.649
Sugar loving, *n* (%)				
No	1	Ref	—	—
Yes	0.994	(0.896-1.101)	-0.119	0.906
Oil loving, *n* (%)				
No	1	Ref	—	—
Yes	1.157	(1.072-1.249)	3.730	<0.001
Salt loving, *n* (%)				
No	1	Ref	—	—
Yes	0.989	(0.932-1.049)	-0.362	0.718
Fatty liver, *n* (%)				
No	1	Ref	—	—
Yes	2.224	(2.168-2.280)	62.430	<0.001
Hypertension, *n* (%)				
No	1	Ref	—	—
Yes	2.373	(2.312-2.435)	65.334	<0.001

Abbreviation: BMI: body mass index.

**Table 3:** Dataset description.

Dataset	Sample distribution	Ratio	Description
Original data	510,411/72,027	7 : 1	Original data with full instances
SMOTE data	510,411/510,411	1 : 1	Dataset is balanced utilizing SMOTE oversampling

**Table 4:** The results of classification algorithms.

Testing criteria	DT	RF	AB	XGB
Confusion matrix	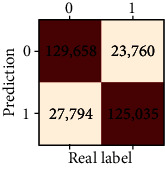	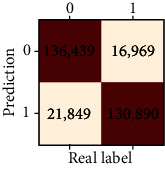	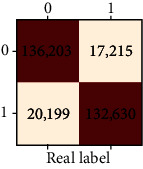	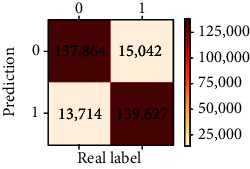
Accuracy	0.832	0.873	0.878	0.906
Precision	0.823	0.862	0.871	0.910
Recall	0.845	0.889	0.888	0.902
*F*‐1	0.834	0.875	0.879	0.906
AUC	0.832	0.947	0.948	0.968

Abbreviations: AUC: the area under the receiver operating characteristic (ROC) curve; DT: decision tree; RF: random forest.

## Data Availability

Data supporting the results of this study can be available by requesting the first author or corresponding author.

## Abbreviations

AUC: Area under the receiver operating characteristic curve
ROC: Receiver operating characteristic curve
NPE: National physical examination
BMI: Body mass index
ML: Machine learning
OR: Odds ratio
DT: Decision tree
LR: Logistic regression
RF: Random forests
WHO: World Health Organization
T2DM: Type II diabetes mellitus
T1DM: Type I diabetes mellitus
ADA: American Diabetes Association.
